# Antibacterial Potential and Chemical Composition of Shiitake Mushroom (*Lentinus edodes* (Berk.) Sing.) Extract Against Pathogenic Bacteria

**DOI:** 10.1155/sci5/6089332

**Published:** 2025-12-18

**Authors:** Waraporn Sutthisa, Pearploy Kamlangmak, Nattaya Srisawad

**Affiliations:** ^1^ Department of Biology, Faculty of Science, Mahasarakham University, Kantarawichai, Mahasarakham Province, 44150, Thailand, msu.ac.th; ^2^ Institute of Molecular Biosciences, Mahidol University, Salaya, Nakhon Pathom Province, 73170, Thailand, mahidol.ac.th

**Keywords:** antimicrobial activity, bioactive compounds, ergosterol, gas chromatography–mass spectrometry (GC–MS), shiitake mushroom

## Abstract

Shiitake mushrooms (*Lentinus edodes* (Berk.) Sing.) have been widely recognized for their bioactive properties, including antimicrobial activity. This study aimed to investigate the antibacterial potential and chemical composition of shiitake mushroom extracts prepared using different solvents (95% ethanol, ethyl acetate, and chloroform). The highest extraction yield (31.16%) was obtained with 95% ethanol. The antimicrobial activity of the extracts was evaluated using the paper disc diffusion method against nine pathogenic bacterial isolates, including Gram‐positive (*Bacillus cereus*, *Staphylococcus aureus*, and *S. aureus* DMST20654) and Gram‐negative (*Escherichia coli*, *E. coli* ATCC25922, *Enterobacter cloacae*, *Pseudomonas aeruginosa*, *Serratia marcescens*, and *Salmonella enterica* serovar *Typhi* ATCC16122) bacteria. Plates were incubated at 37°C for 24 h. All experiments were performed in triplicate. The ethyl acetate extract exhibited the strongest antibacterial activity, with the largest inhibition zone observed for *E. coli* ATCC25922 (30.00 ± 0.00 mm), followed by *S. enterica* serovar *Typhi* ATCC16122 (28.33 ± 2.00 mm). The minimum inhibitory concentration (MIC) and minimum bactericidal concentration (MBC) assays confirmed the superior antibacterial potential of the ethyl acetate extract, particularly against *S. aureus* DMST20654 (MIC = 1.95 mg/mL, MBC = 31.25 mg/mL). Gas chromatography–mass spectrometry (GC–MS) analysis identified key bioactive compounds, including ergosterol (62.38%, %Prob 62.6 in the chloroform extract) and linoleic acid (28.65%, %Prob 56.5 in the ethyl acetate extract), which are known for their antimicrobial properties. The findings highlight the potential of ethyl acetate‐extracted shiitake mushroom compounds as natural antibacterial agents, with applications for food preservation, nutraceuticals, and pharmaceuticals.

## 1. Introduction

Shiitake mushroom (*Lentinus edodes* (Berk.) Sing.) is an aromatic and widely recognized edible mushroom, commonly used in various cuisines, both in fresh and dried forms. It has a soft texture, a distinctive umami flavor, and is frequently included in vegetarian dishes. Beyond its culinary value, shiitake mushrooms have been historically regarded for their medicinal benefits. In traditional Chinese medicine, they are considered a longevity enhancing food, believed to stimulate the immune system, combat bacterial infections, alleviate colds, and improve blood circulation [1]. Additionally, they have been associated with protective effects against heart disease, tumor growth, viral infections, and even snake venom toxicity. Shiitake mushrooms are nutritionally rich, containing essential amino acids, including eritadenine, which aids in cholesterol metabolism, and lentinan, a polysaccharide known for its immune‐boosting properties. Shiitake mushrooms contain a variety of bioactive compounds, including polysaccharides, phenolics, flavonoids, and terpenoids, which contribute to their antimicrobial, antioxidant, and anti‐inflammatory activities [2]. Their antibacterial potential has been extensively studied, with recent research indicating their effectiveness against several pathogenic bacteria. This makes shiitake mushrooms a promising candidate for alternative therapeutic applications, particularly in addressing antibiotic resistance [3].

The increasing prevalence of antibiotic resistant bacteria poses a significant challenge to public health. Pathogens such as *Staphylococcus aureus*, *Escherichia coli*, *Salmonella* spp., and *Pseudomonas aeruginosa* have developed resistance to commonly used antibiotics, necessitating the exploration of new antimicrobial agents [4]. Natural products, particularly those derived from medicinal plants and fungi, have gained attention as alternative sources of antibacterial compounds. Shiitake mushrooms have demonstrated antibacterial potential against both Gram‐positive and Gram‐negative bacteria, suggesting their application for food preservation, pharmaceuticals, and alternative medicine [5]. Previous studies have reported that shiitake mushroom extracts obtained using different solvents exhibit varying degrees of antimicrobial activity. Ethanol, ethyl acetate, and chloroform extractions are commonly used to isolate bioactive compounds from mushrooms, as these solvents facilitate the dissolution of both polar and nonpolar components [6]. The effectiveness of these extracts depends on the presence of specific bioactive compounds, such as lenthionine, a sulfur‐containing antimicrobial compound found in shiitake mushrooms [7]. However, the antimicrobial efficacy of shiitake mushroom extracts against clinically relevant bacterial pathogens remains underexplored.

This study aims to evaluate the antibacterial potential of shiitake mushroom extracts obtained using different solvents and to analyze their chemical composition using gas chromatography–mass spectrometry (GC–MS). The antibacterial activity was assessed through the paper disc diffusion method, and the minimum inhibitory concentration (MIC) and minimum bactericidal concentration (MBC) were determined against selected pathogenic bacteria. The findings from this study should contribute to the development of natural antimicrobial agents from shiitake mushrooms, offering an alternative approach to combating antibiotic‐resistant bacteria.

## 2. Materials and Methods

### 2.1. Shiitake Mushroom Sample Preparation

Fresh shiitake mushrooms were purchased from Makro, Mahasarakham branch, Mueang District, Mahasarakham Province. The mushrooms were washed thoroughly with clean water and allowed to dry completely. Once dried, they were shredded into small, thin pieces and then oven‐dried at 55°C for 3 days using a hot air oven. The dried mushrooms were finely ground using a herb grinder (powder grinder) and stored at 4°C until further analysis.

### 2.2. Preparation of Shiitake Mushroom Extract

Shiitake mushroom extracts were prepared using three different solvents: 95% ethanol, ethyl acetate, and chloroform. A total of 50 g of finely ground shiitake mushroom powder was placed into three separate 1,000 mL flasks. Each flask was then filled with 450 mL of one of the solvents (95% ethanol, ethyl acetate, or chloroform). The flasks were covered with cotton stoppers and sealed with aluminum foil. The extraction process was carried out at room temperature using an orbital shaker set at 150 rpm for 48 h. After extraction, the mushroom powder residues were removed through sequential filtration using muslin cloth, a tea bag, and Whatman No. 1 filter paper until a clear extract was obtained. The filtered extracts were then concentrated using a rotary evaporator (Buchi Vacuum Pump set of 215+V‐700/V‐855**,** Switzerland) with a vacuum system at 45°C to remove the solvents. The extracts were further dried to ensure complete solvent evaporation, leaving behind only the crude extracts. The obtained extracts were weighed, and their physical characteristics such as color, odor, and viscosity were observed for each solvent (95% ethanol, ethyl acetate, and chloroform). Finally, the percentage yield (% yield) of each crude extract was calculated using the following formula [8]:

(1) % yield=weight of extractgweight of dried plant materialg×100.

The extracts were stored in sterile amber bottles at −20°C until further testing on pathogenic bacteria.

### 2.3. Preparation of Bacterial Inoculum

The test bacteria included both Gram‐positive and Gram‐negative species. The Gram‐positive bacteria used were *Bacillus cereus*, *Staphylococcus aureus*, and *Staphylococcus aureus* DMST 20654. The Gram‐negative bacteria included *Escherichia coli*, *Escherichia coli* ATCC 25922, *Enterobacter cloacae*, *Pseudomonas aeruginosa*, *Serratia marcescens*, and *Salmonella enterica* serovar *Typhi* ATCC 16122. All bacterial strains were kindly provided by the Microbiology Laboratory, Department of Biology, Faculty of Science, Mahasarakham University. The bacterial cultures were grown on nutrient agar (NA) at 37°C for 18–24 h. Colonies were then carefully scraped and suspended in normal saline solution. The turbidity of the bacterial suspension was adjusted to match the McFarland standard No. 0.5, corresponding to a bacterial density of approximately 1.5 × 10^8^ CFU/mL.

### 2.4. Evaluation of the Antimicrobial Effect of Shiitake Mushroom Extract on Pathogenic Bacteria Using the Paper Disc Diffusion Method

Test bacteria were cultured to the desired concentration and evenly spread on NA using a sterile cotton swab. After allowing the surface to dry for 3–5 min, sterile paper discs were placed on the medium. Shiitake mushroom extract was prepared using 95% ethanol, ethyl acetate, and chloroform, with a final concentration of 250 mg/mL. 20 μL of the extract was applied to each disc, while streptomycin (3 mg/mL) served as a positive control and sterile distilled water as a negative control. The plates were incubated at 37°C for 24 h. All tests were performed in triplicate (*n* = 3). After incubation, the inhibition zone was measured by determining the diameter of the clear zone from one edge to the opposite edge through the center of the disc, and the results were recorded in millimeters.

### 2.5. Determination of the MIC and MBC

The MIC of shiitake mushroom extracts was determined following the preliminary efficacy test. Extracts obtained using ethyl acetate and chloroform, which exhibited antibacterial activity, were selected for further analysis. These extracts were diluted in sterile distilled water using a two‐fold serial dilution to obtain final concentrations of 500, 250, 125, 62.5, 31.25, 15.62, 7.81, 3.91, 1.95, 0.98, and 0.49 mg/mL. A total of 100 μL of each diluted extract was added to wells 1–11 of a 96‐well plate, while well 12 served as a negative control containing only Mueller Hinton broth (MHB). Streptomycin (3 mg/mL) was used as a positive control. Test bacteria were prepared at a concentration of 1.5 × 10^8^ CFU/mL and diluted 1:100 in liquid medium to obtain approximately 10^6^ CFU/mL, which was used as the starter culture. Then, 100 μL of the bacterial suspension was added to wells 1–12, and the plate was incubated at 37°C for 24 h. After incubation, bacterial growth was assessed by measuring turbidity at 600 nm using an ASYS UVM 340 microplate reader.

Following MIC determination, the MBC was assessed by subculturing 10 μL aliquots from wells showing no visible growth (and from the first well showing turbidity) onto nutrient agar plates. The plates were then incubated at 37°C for 24 h. After incubation, the MBC was recorded as the lowest concentration of extract that showed no bacterial growth on the agar surface, indicating complete killing of the microorganism.

### 2.6. Study of Chemical Composition of Shiitake Mushroom Extract by GC–MS

The chemical composition of shiitake mushroom extracts was analyzed using GC–MS. Samples of shiitake mushroom extracts obtained using 95% ethanol, ethyl acetate, and chloroform were each weighed at 50 mg, dissolved in 1 mL of methanol, and filtered through a 0.45‐micron membrane before analysis. Analyses were performed on an Agilent 7890A GC system coupled to a 7000B MS. Separation was achieved on a DB‐5MS capillary column (30 m × 0.25 mm i.d., 0.25 μm film thickness). The injection volume was 1.0 μL (split ratio 10:1), with helium as the carrier gas at a constant flow rate of 1.0 mL min^−1^. The oven temperature program was as follows: initial temperature 40°C (held 2 min), increased at 10°C min^−1^–280°C, and held for 5 min, giving a total run time of approximately 35 min per sample. The ion source temperature was 230°C, and the transfer line was maintained at 280°C under the electron‐impact (EI) ionization mode at 70 eV. Mass spectra were recorded in the range m/z 35–550 with a solvent delay of 2 min. Chromatograms and mass spectra were interpreted by comparing retention times and spectral fragmentation patterns with the NIST MS Search 2.0 Library. Compounds were reported with their matching probability (%Probability), indicating the confidence of spectral identification. Compounds showing low probability values or those potentially derived from solvents were noted and interpreted cautiously to avoid misrepresentation. Although blank solvent injections were not performed, care was taken to critically evaluate the spectra for potential solvent‐derived peaks, and any compounds suspected to originate from the extraction solvents were noted and interpreted cautiously to ensure accurate representation of the extract composition. All identified compounds and their %Probability are listed in Table 1.

**Table 1:** Chemical composition of Shiitake mushroom extracts analyzed by gas chromatography–mass spectrometry (GC–MS).

No.	RT	Name	Chloroform extract		Ethyl acetate extract		95% ethanol extract	
			% relative peak areas	%probability	% relative peak areas	%probability	% relative peak areas	%probability
1	3.101	1‐Propanol	—	—	0.79	88.4	—	—
2	3.210	Ammonium acetate	—	—	—	—	49.61	79.7
3	3.318	Acetic acid	—	—	7.48	83.1	—	—
4	3.342	Propanoic acid, ethyl ester	—	—	—	—	0.35	96.7
5	3.361	Acetic acid, propyl ester	—	—	—	—	0.26	90.9
6	4.031	Diethyl carbonate	0.13	95.3	—	—	—	—
7	4.514	1‐Butanamine, 3‐methyl‐	—	—	1.36	79.2	—	—
8	5.175	Acetic acid, butyl ester	—	—	—	—	1.20	92.0
9	6.490	Urethane	—	—	0.46	97.7	—	—
10	7.565	1,2‐Propanediol, 2‐acetate	—	—	—	—	0.14	94.8
11	7.991	Dimethyl sulfone	0.04	92.8	—	—	—	—
12	9.405	1‐Butanamine, N‐butyl‐	0.06	68.8	—	—	—	—
13	10.067	1,2‐Ethanediol, diacetate	—	—	—	—	0.18	88.8
14	10.796	1,2‐Propanediol, diacetate	—	—	—	—	0.05	94.0
15	10.813	2H‐Pyran‐2‐one, 5,6‐dihydro‐	0.04	67.0	—		—	—
16	10.864	Acetamide, N‐(2‐methylpropyl)‐	—	—	—	—	0.23	91.8
17	10.894	1‐Butanamine, 2‐methyl‐N‐(2‐methylbutylidene)‐	0.14	90.7	—	—	—	—
18	12.115	2‐Pyrrolidinone	—	—	0.38	94.2	—	—
19	12.387	Benzenediamine	0.20	88.7	0.34	81.4	—	—
20	12.615	Phenylethyl alcohol	0.14	78.2	—	—	—	—
21	12.760	1,2,3‐Propanetriol, 1‐acetate	—	—	—	—	3.07	97.3
22	13.240	N‐(3‐Methylbutyl)acetamide	0.14	95.6	—	—	0.55	92.6
23	13.873	N‐Methyliminopropylbenzene	0.21	76.5	—	—	—	—
24	14.169	Methyl salicylate	—	—	0.37	74.7	—	—
25	19.646	Acetamide, N‐(2‐phenylethyl)‐	0.65	86.7	—	—	1.19	93.9
26	24.930	Lidocaine	—	—	1.16	57.3	—	—
27	25.765	n‐Hexadecanoic acid	0.44	71.1	2.35	82.3	—	—
28	26.020	Hexadecanoic acid, ethyl ester	0.06	71.1	—		—	—
29	27.778	Methyl stearate	—	—	—	—	0.11	76.4
30	27.957	Palmidrol	0.31	79.7	—	—	—	—
31	28.156	Linoleic acid	1.04	51.3	28.65	56.5	—	—
32	28.637	Hexadecanamide	0.07	71.0	0.37	73.7	0.61	69.6
33	31.089	9‐Octadecenamide, (Z)‐	0.10	65.7	2.32	77.7	4.77	92.7
34	31.695	Pentadecanoic acid, 2‐hydroxy‐1‐(hydroxymethyl)ethyl ester	—	—	0.45	92.1	—	—
35	33.163	Glycerol β‐palmitate	0.58	60.1	5.13	58.0	—	—
36	36.879	13‐Docosenamide, (Z)‐	0.22	87.4	—	—	—	—
37	39.204	Dehydroergosterol 3,5‐dinitrobenzoate	—	—	—	—	0.62	50.4
38	42.546	Ergosterol	62.38	62.6	8.10	64.9	14.89	64.9
39	43.046	Neoergosterol	1.47	84.1	—	—	—	—

*Note:* Compounds were identified by comparing mass spectra with the NIST MS Search 2.0 Library. The % probability represents the confidence level of spectral matching between experimental and reference spectra. Only compounds with a library match of ≥ 50% were included. Peaks with match scores below 50% and those suspected to originate from extraction solvents were excluded to avoid misinterpretation. Values are expressed as relative percentage of total detected peak area.

## 3. Results and Discussion

### 3.1. Sample Preparation of Shiitake Mushroom

A total of 1,982.84 g of fresh shiitake mushrooms were used for sample preparation. After drying, the weight of the dried shiitake mushrooms was recorded as 360.50 g. The dried mushrooms were then ground into a fine powder for further testing.

### 3.2. Extraction of Shiitake Mushroom

The yield of extraction of shiitake mushroom (*Lentinus edodes*) varied depending on the solvent used, with 95% ethanol providing the highest yield (31.16%), followed by chloroform (13.08%) and ethyl acetate (10.20%). The differences in extraction yield can be attributed to the polarity of the solvents and the solubility of bioactive compounds present in the mushroom. Ethanol, being a polar solvent, is highly effective in extracting a wide range of hydrophilic and moderately lipophilic compounds, including polysaccharides, flavonoids, and phenolics, which contribute to its high yield [9]. The orange‐brown coloration and smooth texture of the ethanol extract further suggest the presence of these bioactive compounds, which are known for their antimicrobial and antioxidant properties [2]. On the other hand, ethyl acetate, a semipolar solvent, yielded the lowest extraction efficiency (10.20%). This is likely due to its selective solubility for moderately polar compounds, such as certain flavonoids and terpenoids, while being less effective at extracting polysaccharides and other hydrophilic constituents. The resulting extract exhibited a sticky and less smooth texture, suggesting a different composition compared to ethanol extracts [10]. Chloroform, a nonpolar solvent, resulted in a 13.08% yield and produced a dark brown extract with a fine, smooth texture. This indicates its ability to extract nonpolar compounds such as lipids, sterols, and some terpenoids, which are commonly present in mushrooms. The yield obtained with chloroform is higher than that of ethyl acetate, suggesting the presence of a considerable proportion of lipophilic compounds in shiitake mushrooms [11]. Overall, the choice of solvent significantly influenced the extract yield and composition. Ethanol was the most effective in obtaining a higher yield, likely due to its ability to solubilize a broad spectrum of bioactive compounds. These findings align with previous studies, which indicate that ethanol is commonly used for extracting bioactive components from medicinal mushrooms due to its efficiency and safety for potential pharmaceutical applications [12].

### 3.3. The Antimicrobial Activity of Shiitake Mushroom Extract on Pathogenic Bacteria Using the Paper Disc Diffusion Method

The results of the paper disc diffusion method demonstrated that the antimicrobial activity of shiitake mushroom extracts varied significantly depending on the solvent used for extraction.

Statistical analysis using the LSD method revealed significant differences both among extracts for a given pathogen (indicated by different lowercase (small) letters within each row) and among pathogens for a given extract (indicated by different uppercase (capital) letters within each column) (Table 2). The ethanolic extract did not exhibit any inhibitory effect against the tested bacterial isolates, whereas ethyl acetate and chloroform extracts demonstrated notable antibacterial activity (Table 2). The ethyl acetate extract exhibited the highest antibacterial activity, with inhibition zones ranging from 17.83 ± 0.76 mm (*S. aureus* DMST 20654) to 30.00 ± 0.00 mm (*E. coli* ATCC 25922). The broad‐spectrum activity of this extract against both Gram‐positive and Gram‐negative bacteria suggests the presence of bioactive compounds with strong antibacterial properties. Similar results have been reported in previous studies, where ethyl acetate extracts of medicinal mushrooms demonstrated superior antibacterial activity compared to extracts obtained with other solvents [13, 14]. The high efficacy of the ethyl acetate extract may be attributed to its ability to extract nonpolar to moderately polar bioactive compounds, such as phenolics, flavonoids, and terpenoids, which have been identified as key antimicrobial agents in mushrooms [15].

**Table 2:** Inhibitory activity of shiitake mushroom extracts against pathogenic bacteria by paper disc diffusion.

Pathogen	Inhibition zone (mm)			
	Chloroform extract	Ethyl acetate extract	95% ethanol extract	Streptomycin
*Bacillus* cereus	12.88 ± 0.39^cDE^	20.22 ± 1.58^bEF^	0.00 ± 0.00^d^	33.67 ± 1.53^aAB^
*Staphylococcus aureus*	12.33 ± 0.33^cE^	21.44 ± 2.59^bDE^	0.00 ± 0.00^d^	34.00 ± 1.00^aA^
*Staphylococcus aureus* DMST20654	13.00 ± 0.00^cD^	17.83 ± 0.76^bG^	0.00 ± 0.00^d^	31.33 ± 1.53^aCDE^
*Escherichia coli*	16.44 ± 0.51^cAB^	21.55 ± 0.51^bDE^	0.00 ± 0.00^d^	30.00 ± 1.00^aE^
*Escherichia coli* ATCC 25922	17.00 ± 0.00^cA^	30.00 ± 0.00^bA^	0.00 ± 0.00^d^	30.33 ± 1.53^aDE^
*Enterobacter cloacae*	14.33 ± 0.58^cC^	19.33 ± 0.58^bF^	0.00 ± 0.00^d^	32.33 ± 2.00^aABC^
*Pseudomonas aeruginosa*	9.10 ± 0.17^cF^	22.66 ± 0.34^bD^	0.00 ± 0.00^d^	32.00 ± 1.73^aBCD^
*Serratia marcescens*	7.88 ± 1.07^bG^	26.77 ± 1.26^aC^	0.00 ± 0.00^c^	26.67 ± 1.53^aF^
*Salmonella enterica* serovar *Typhi* ATCC16122	16.33 ± 0.58^cB^	28.33 ± 2.08^bB^	0.00 ± 0.00^d^	32.00 ± 2.00^aBCD^

*Note:* Values are expressed as mean ± standard deviation (mm; *n* = 3). For each row (within a pathogen), different lowercase (small) letters (a, b, c, d) indicate significant differences among extracts (chloroform, ethyl acetate, ethanol, and streptomycin) according to the least significant difference (LSD) test at *p* < 0.05. For each column (within an extract type), different uppercase (capital) letters (A, B, C, etc.) indicate significant differences among bacterial species according to the LSD test at *p* < 0.05.

The chloroform extract also displayed antibacterial activity against all tested bacterial isolates but with smaller inhibition zones compared to the ethyl acetate extract. The inhibition zones ranged from 7.88 ± 1.07 mm (*S. marcescens*) to 17.00 ± 0.00 mm (*E. coli* ATCC 25922). Chloroform is known to extract nonpolar compounds such as fatty acids, steroids, and certain terpenes, which may contribute to the observed antimicrobial activity. Previous studies have shown that chloroform extracts of shiitake mushrooms possess antibacterial properties, though their efficacy is generally lower than that of ethyl acetate extracts [16]. Interestingly, the ethanolic extract showed no inhibitory activity against any of the tested bacteria. This observation can be explained by the chemical composition revealed through GC–MS and related phytochemical analyses: ethanol preferentially extracts polar compounds, such as polysaccharides, sugar alcohols, and certain organic acids, which are abundant in *Lentinula edodes* but are primarily associated with immunomodulatory or antioxidant effects rather than direct antimicrobial activity. For example, the GC–MS analysis of mushroom ethanolic extracts has reported high contents of compounds like isosorbide and dianhydromannitol, along with phenolic acids that do not necessarily confer strong antibacterial action [16]. Therefore, the lack of inhibition zones in the ethanol extract is consistent with the absence or low concentration of bioactive compounds with direct antibacterial properties. Among the tested bacteria, *E. coli* ATCC 25922 and *S. enterica* serovar *Typhi* ATCC 16122 were the most susceptible to the shiitake mushroom extracts, particularly those obtained with ethyl acetate. The strong inhibition of *E. coli* aligns with previous studies that have reported the effectiveness of mushroom extracts against members of the Enterobacteriaceae family [17]. The notable susceptibility of *S. enterica* serovar *Typhi* suggests that shiitake mushroom extracts could be explored as a potential natural antimicrobial agent against foodborne pathogens.

Overall, these findings highlight the importance of solvent selection in extracting bioactive compounds with antimicrobial properties from shiitake mushrooms. Ethyl acetate was the most effective solvent for obtaining antimicrobial compounds, likely due to its ability to extract phenolic and flavonoid compounds responsible for bacterial inhibition. Integrating the chemical profile data, it is evident that ethanol, despite efficiently extracting polar constituents, does not yield sufficient quantities of direct antibacterial agents. Further studies are needed to identify the specific active compounds and their mechanisms of action. These results also suggest the potential application of shiitake mushroom extracts for food preservation and alternative therapeutic strategies against bacterial infections.

### 3.4. The MIC and MBC

The determination of the MIC and MBC of shiitake mushroom extracts revealed notable differences in antibacterial activity between the chloroform and ethyl acetate extracts. The ethyl acetate extract exhibited significantly stronger antibacterial effects than the chloroform extract, as evidenced by lower MIC and MBC values against various bacterial pathogens (Table 3). The chloroform extract demonstrated limited antibacterial activity, with MIC values ranging from 31.25 mg/mL (*S. enterica* serovar *Typhi* ATCC16122) to 250 mg/mL (*S. aureus*). Notably, *P. aeruginosa* had the lowest MIC (125 mg/mL) but an MBC exceeding 250 mg/mL, indicating that the bactericidal concentration was not reached at the highest concentration tested. Similarly, *B. cereus* and *S. aureus* showed MIC values of 250 mg/mL, with MBC values greater than 250 mg/mL. These results indicate that, for these pathogens, the chloroform extract exerted a bacteriostatic effect rather than a bactericidal effect at the concentrations tested. These findings are consistent with previous research suggesting that chloroform extracts generally exhibit moderate antibacterial properties, likely due to their ability to extract nonpolar bioactive compounds with limited solubility in aqueous environments [18]. In contrast, the ethyl acetate extract exhibited significantly stronger antibacterial activity. The lowest MIC value was observed for *S. aureus* DMST20654 (1.95 mg/mL), with an MBC of 31.25 mg/mL, indicating potent bactericidal effects. *B. cereus*, *E. coli*, and *S. enterica* serovar *Typhi* ATCC16122 also displayed strong susceptibility to the ethyl acetate extract, with MIC values of 3.91 mg/mL. These results align with previous studies that report ethyl acetate extracts as highly effective against bacterial pathogens due to their ability to extract polar and semipolar bioactive compounds such as flavonoids, phenolics, and terpenoids [19, 20].

**Table 3:** Minimum inhibitory concentration (MIC) and minimum bactericidal concentration (MBC) of shiitake mushroom extracts.

Pathogen	Chloroform extract		Ethyl acetate extract	
	MIC (mg/mL)	MBC (mg/mL)	MIC (mg/mL)	MBC (mg/mL)
*Bacillus* cereus	250	> 250	3.91	7.81
*Staphylococcus aureus*	250	> 250	7.81	15.62
*Staphylococcus aureus* DMST20654	125	> 250	1.95	31.25
*Escherichia coli*	250	250	3.91	31.25
*Escherichia coli* ATCC 25922	250	> 250	7.81	31.25
*Enterobacter cloacae*	250	> 250	7.81	31.25
*Pseudomonas aeruginosa*	125	> 250	7.81	7.81
*Serratia marcescens*	> 250	> 250	7.81	15.62
*Salmonella enterica* serovar *Typhi* ATCC16122	31.25	125	3.91	15.62

*Note:* Values represent endpoint concentrations determined by two‐fold serial dilution assays. All tests were performed in triplicate; since all replicates gave the same endpoint concentration, standard deviation values are not applicable. “> 250 mg/mL” indicates that the MBC was not achieved at the highest concentration tested, and therefore the extract exhibited a bacteriostatic effect rather than a bactericidal effect for these pathogens.

When compared with other natural antimicrobials, the MIC values of the shiitake ethyl acetate extract in this study are within a promising and often superior range. For example, ethyl acetate extracts of *Ganoderma lucidum* have been reported to inhibit *S. aureus* at MIC values of 2–4 mg/mL [21], while ethanolic extracts of *Lentinus sajor-caju* showed MICs against *S. aureus* ranging from 4 to 8 mg/mL [22]. Similarly, plant‐derived compounds such as *Curcuma longa* (turmeric) ethanolic extracts typically show MIC values against *S. aureus* in the range of 2–16 mg/mL depending on extraction methods [23]. Additional studies on mushroom extracts also support this comparison. Ethanol extracts of *Lentinula edodes* have been reported to exhibit MICs between 5.1 and 6.01 mg/mL against pathogens such as *Klebsiella pneumoniae*, *S. aureus*, *Enterococcus faecalis,* and *Acinetobacter baumannii*, while *Agaricus bisporus* extracts showed MICs between 5.8 and 9.54 mg/mL [16]. Furthermore, crude mycelial extract of the entomopathogenic fungus *Polycephalomyces nipponicus* was active against methicillin‐resistant *S. aureus* (MRSA) with a reported MIC of 3 mg/mL and was shown to disrupt energy metabolism and translation [24]. Likewise, ethanolic fractions of wild mushrooms such as *Meripilus giganteus* and *Pleurotus flabellatus* exhibited strong antimicrobial activity, whereas their ethyl acetate fractions showed comparatively weaker effects [25]. In comparison, the MIC of the shiitake ethyl acetate extract observed in this study (1.95 mg/mL against *S. aureus* DMST20654) is notably lower than many of the reported values for other fungal extracts and is at least comparable to or stronger than many plant‐based natural antimicrobials. This underscores the strong antibacterial potential of shiitake ethyl acetate extract and highlights its relevance for further development as a natural antibacterial agent.

### 3.5. Chemical Composition by Gas Chromatography–Mass Spectrometry (GC–MS) and Antimicrobial Activity of Shiitake Mushroom Extracts

Gas Chromatography–Mass Spectrometry (GC–MS) analysis of shiitake mushroom extracts revealed a complex profile of bioactive compounds with potential antimicrobial properties. The identified compounds varied among the chloroform, 95% ethanol, and ethyl acetate extracts, reflecting the influence of solvent polarity on extraction efficiency. The identification of compounds was based on spectral matching with the NIST MS Search 2.0 Library, and similarity indices are presented as %Probability values in Table 1 (Figure 1). Compounds showing lower similarity probabilities or possible solvent origins were noted and interpreted cautiously. A 50% NIST library‐match threshold was applied for preliminary compound identification. Although higher thresholds (≥ 70%) increase spectral‐matching confidence, many natural fungal metabolites—particularly sterols, long‐chain fatty acids, and terpenoid derivatives—often produce moderate match scores due to limited reference spectra, structural similarity among analogs, and characteristic fragmentation patterns. Therefore, a 50% cutoff provides a practical and widely used approach in untargeted GC–MS profiling of natural product extracts, allowing the retention of biologically relevant components such as ergosterol and linoleic acid derivatives. All reported compounds were further supported by diagnostic fragment ions, expected retention behavior, and consistency with chemical profiles previously described in shiitake mushrooms.

Figure 1Chromatograms of chemical components in shiitake mushroom extracts obtained by gas chromatography–mass spectrometry (GC–MS). (a) Chloroform extract; (b) 95% ethanol extract; (c) ethyl acetate extract.

**Figure:**
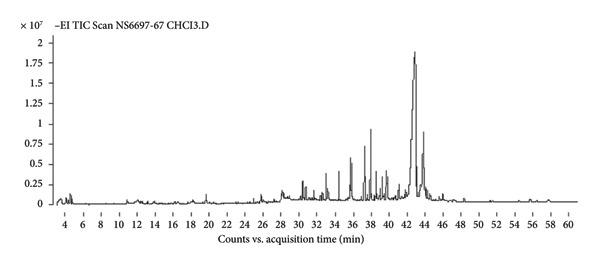
(a)

**Figure:**
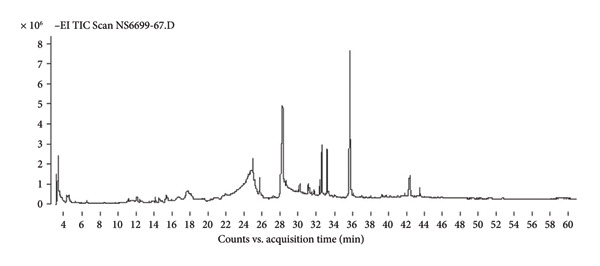
(b)

**Figure:**
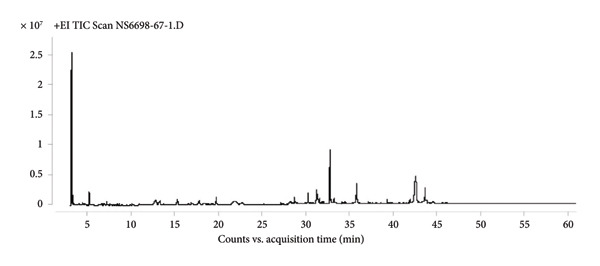
(c)

Dominant compounds: ergosterol, the most abundant compound in all extracts especially the chloroform extract (62.38%, %Prob 62.6) is a critical fungal sterol known for its antimicrobial properties. Ergosterol plays a crucial role in fungal cell membrane integrity, and its presence in high concentrations may contribute to the antimicrobial activity of shiitake mushroom extracts [26]. Additionally, ergosterol has been reported to inhibit the growth of pathogenic bacteria, particularly Gram‐positive species [27].

Amino acid derivatives: the detection of N,N‐dimethylaminoethanol and acetamide, N‐(2‐phenylethyl), in the extracts suggests potential contributions to antimicrobial activity. N,N‐Dimethylaminoethanol is a precursor to choline, which plays a role in microbial membrane integrity, and its presence may affect microbial growth. Additionally, 2,3‐butanediol, a fermentation‐derived compound found in the ethanol and ethyl acetate extracts, has been shown to inhibit bacterial quorum sensing, thereby reducing bacterial virulence [28]. Terpenoids: squalene, a bioactive terpenoid found in all extracts, has been reported to possess antimicrobial and antioxidant properties [29]. Squalene has been shown to interfere with bacterial cell wall synthesis, leading to growth inhibition. The presence of methyl salicylate, another terpenoid compound, further supports the antimicrobial potential of the extracts, as it is known for its antibacterial and anti‐inflammatory effects [30].

The differences in extracted compounds based on solvent type are significant. The ethanol extract contained higher levels of linoleic acid and ergosterol, suggesting that ethanol is particularly effective in extracting lipophilic (fat‐soluble) antimicrobial compounds. In contrast, the ethyl acetate extract contained higher concentrations of acetic acid, ammonium acetate, and alcohol derivatives, which may contribute to antimicrobial properties by disrupting bacterial metabolism [31].

These findings emphasize the importance of solvent selection in optimizing the extraction of antimicrobial compounds from shiitake mushrooms. The presence of multiple bioactive compounds in different extracts highlights the potential of shiitake mushrooms as a natural source of antimicrobial agents, supporting their applications for food preservation, nutraceuticals, and pharmaceuticals [32]. The GC–MS analysis confirmed that shiitake mushroom extracts contain a variety of bioactive compounds with known antimicrobial properties. Ergosterol, linoleic acid, and squalene are among the most abundant and significant compounds, suggesting their central role in antimicrobial activity. The variation in composition across different solvents highlights the importance of solvent selection in extracting specific bioactive compounds. These findings reinforce the potential of shiitake mushrooms as an alternative antimicrobial agent with broad‐spectrum applications for food preservation and medicine.

Although ergosterol was the most abundant compound in the chloroform extract (62.38%, %Prob 62.6), this extract exhibited relatively weaker antimicrobial activity compared to the ethyl acetate extract. This indicates that antimicrobial efficacy cannot be attributed solely to the concentration of a single compound. Instead, the overall activity is likely driven by synergistic interactions among multiple constituents. Several of the major compounds identified have documented antibacterial mechanisms. Ergosterol, although primarily a fungal sterol, can insert into bacterial membranes, disrupting lipid packing and increasing permeability; studies have shown that ergosterol inhibits the growth of *S*. *aureus* and other Gram‐positive bacteria by compromising membrane integrity [33]. Sterol compounds identified in the shiitake extracts, particularly ergosterol and γ‐ergostenol, are known to play important roles in antimicrobial mechanisms. Previous studies reported that sterols isolated from medicinal mushrooms, such as those from *Ganoderma atrum*, exhibited significant antimicrobial effects against both Gram‐positive and Gram‐negative bacteria while also providing antioxidant protection to mammalian cells [34]. These findings support the view that sterols detected in our shiitake extracts, such as ergosterol and γ‐ergostenol, may contribute to antimicrobial activity by disrupting bacterial membranes and interfering with essential cellular processes [35]. It is also important to note that not all sterol or steroid compounds demonstrate broad‐spectrum antibacterial effects. For example, a study investigating the antibacterial effects of various steroids found that only corticosterone (MIC 32 μg/mL against *Pasteurella multocida*) and β‐sitosterol (MIC 32 μg/mL against *S. aureus*) showed measurable activity, whereas other steroids, including progesterone, estrone, and stigmasterol, had no effect on the tested bacteria [36]. These findings highlight that antibacterial activity among steroidal compounds is highly structure‐dependent and not universal. In the context of our study, this supports the interpretation that the antimicrobial properties of mushroom‐derived sterols are compound‐specific and likely act synergistically with other bioactive constituents rather than representing an inherent characteristic of all steroids. Likewise, linoleic acid and other unsaturated fatty acids exhibit strong antimicrobial action through membrane disruption and inhibition of fatty acid synthesis; linoleic acid, for instance, has been shown to inhibit *S. aureus* growth and reduce toxin production by targeting cell membranes and interfering with energy metabolism [34].

Finally, solvent‐dependent bioavailability and extractability play key roles [36, 37]. Ethyl acetate, with intermediate polarity, may co‐extract amphiphilic compounds that facilitate the incorporation of sterols and fatty acids into bacterial membranes, thereby enhancing antimicrobial effects. In contrast, the chloroform extract, despite its high ergosterol content, may lack such complementary compounds or may extract constituents that are less bioavailable in aqueous environments. Thus, the observed antimicrobial activity is best explained by a combination of compound profile, solvent polarity, and synergistic interactions rather than by the presence of a single dominant compound [38, 39].

## 4. Conclusions

This study demonstrates the antibacterial potential of shiitake mushroom extracts against various pathogenic bacteria, with ethyl acetate proving to be the most effective solvent for extracting bioactive compounds with antimicrobial properties. The identification of key compounds such as ergosterol and linoleic acid further supports the antimicrobial potential of the extract. The variation in antimicrobial activity based on solvent type emphasizes the critical role of extraction methods in optimizing bioactive compound recovery. The results suggest that ethyl acetate extracts of shiitake mushrooms could serve as natural antimicrobial agents with applications for food preservation and alternative therapeutic strategies. Further research is needed to isolate specific active compounds and explore their mechanisms of action in antimicrobial applications.

## Conflicts of Interest

The authors declare are no conflicts of interest.

## Funding

This research project was financially supported by Mahasarakham University.

## Data Availability

The data that support the findings of this study are available from the corresponding author upon reasonable request.
